# Transbronchial and transesophageal fine-needle aspiration using a single ultrasound bronchoscope in the diagnosis of locoregional recurrence of surgically-treated lung cancer

**DOI:** 10.1186/s12890-017-0388-4

**Published:** 2017-02-28

**Authors:** José Sanz-Santos, Pere Serra, Felipe Andreo, Mohamed Torky, Carmen Centeno, Teresa Morán, Enric Carcereny, Esther Fernández, Samuel García-Reina, Juan Ruiz-Manzano

**Affiliations:** 1 Pulmonology Department, Hospital Germans Trias i Pujol, Carretera de Canyet S/N. 08916, Badalona, Barcelona Spain; 2 Department de Medicina. Universitat Autònoma de Barcelona, Bellaterra, Barcelona Spain; 3 Catalan Institute of Oncology, Badalona, Barcelona Spain; 4 Thoracic Surgery Department, Hospital Germans Trias i Pujol, Badalona, Barcelona Spain

**Keywords:** Lung cancer, Recurrence, Surgically-treated, Endobronchial Ultrasound, Transbronchial needle aspiration, Endoscopic ultrasound, Fine needle aspiration

## Abstract

**Background:**

The present study sought to evaluate the usefulness of EBUS-TBNA in the diagnosis of locoregional recurrence of lung cancer in a cohort of lung cancer patients who were previously treated surgically, and describe our initial experience of EUS-B-FNA in this clinical scenario.

**Methods:**

We retrospectively studied the clinical records of all patients with a previous surgically-treated lung cancer who were referred to our bronchoscopy unit after suspicion of locoregional recurrence. The diagnostic sensitivity, specificity, positive predictive value (PPV), negative predictive value (NPV) and overall accuracy of EBUS-TBNA for the diagnosis of locoregional recurrence were evaluated.

**Results:**

Seventy-three patients were included. EBUS-TBNA confirmed malignancy in 40 patients: 34 confirmed to have locoregional recurrence, six had metachronous tumours. Of the 33 patients with non-malignant EBUS-TBNA; 2 had specific non-malignant diseases, 26 underwent radiological follow up and 5 patients underwent surgery. Of the 26 patients who had radiological follow up; 18 remained stable, three presented thoracic radiological progression and 5 presented extrathoracic progression. Of the 5 patients who underwent surgery; 3 had metachronous tumours, one confirmed to be a true negative and one presented nodal invasion. Seven patients underwent EUS-B-FNA, four of them confirmed to have recurrence. The sensitivity, specificity, NPV, PPV and overall accuracy of EBUS-TBNA for the diagnosis of locoregional recurrence were 80.9, 100, 69.2, 100 and 86.6% respectively.

**Conclusions:**

EBUS-TBNA is an accurate procedure for the diagnosis of locoregional recurrence of surgically-treated lung cancer. EUS-B-FNA combined with EBUS-TBNA broads the diagnostic yield of EBUS-TBNA alone.

## Background

Lung cancer is the leading cause of cancer-related mortality worldwide [1]. Surgery resection with a curative intent is the most effective treatment for early stage non-small- cell lung cancer (NSCLC). However, even after complete surgical resection, the rate of recurrence for stages I to III of NSCLC range from 30 to 70% [2] with a high incidence of recurrence during the first 2 years. Based on this concern, published practice guidelines recommend multidisciplinary clinical and radiographic follow up of patients with resected lung cancer [3]. More specifically, the American College of Chest Physicians recommends that in patients who underwent a curative-intent surgical resection of a NSCLC, a chest computer tomography (CT) should be performed every 6 months for the first 2 years after resection and every year thereafter. In many cases, surgical treatment must be combined with adjuvant chemotherapy and/or radiotherapy which can cause inflammation and fibrosis of the mediastinum. Furthermore, most of lung cancer patients are smokers who may have Chronic Obstructive Pulmonary Disease (COPD) or other inflammatory lung disorders which may result in infectious complications. These circumstances such as inflammation, fibrosis and infectious complications could be presented as both lung parenchymal abnormalities and mediastinal nodal enlargement in thoracic CT that can be misdiagnosed as regional recurrence [4]. Thereby, mediastinal and/or hilar nodal enlargement in the thoracic CT during follow up are common features that usually represent a challenge for the clinician. Positron emission tomography with computer tomography (PET/CT) has been used as a diagnostic tool for recurrence, with a high sensitivity value [5, 6]. However, both CT and PET/CT have shown high false-positive rates and therefore histological confirmation is mandatory to rule out regional recurrence.

Mediastinoscopy has been demonstrated to be useful in the assessment of recurrence in patients with previous surgically-treated lung cancer [7]. However, mediastinoscopy becomes more difficult, unsafe and useless after previous thoracic surgery, especially in patients who have been treated with induction radiotherapy. Furthermore, recurrence can lay on hilar nodes that are not amenable for biopsy by means of mediastinoscopy.

Endobronchial ultrasound-guided transbronchial needle aspiration (EBUS-TBNA) is a minimum invasive procedure currently proposed as the first choice in the mediastinal nodal staging of lung cancer [8]. Adding endoscopic ultrasound fine needle aspiration (EUS-FNA) to EBUS-TBNA has revealed higher diagnostic yield than using EBUS-TBNA alone, due to the complementary access for different nodal stations by each technique [9]. The evaluation of mediastinal nodes from the esophagus using a convex probe EBUS (EUS-B-FNA) associated to EBUS-TBNA has proven to be useful, with figures similar to those using combination of EUS and EBUS-TBNA [10]. The applications of EUS-B-FNA further than lung cancer staging have been barely investigated [11–13].

Some previous studies have demonstrated the usefulness of EBUS-TBNA in the diagnosis of recurrence in patients with previously treated lung cancer [14–18]. However, some of these studies included short series of subjects or included patients not surgically treated. The aim of our study was to evaluate the usefulness of EBUS-TBNA in the diagnosis of locoregional lung cancer recurrence in a larger cohort of subjects entirely composed of surgically-treated patients and describe our initial experience of EUS-B-FNA in this clinical scenario.

## Methods

### Patients

We conducted a single-center, retrospective study that included all patients with a previous surgically-treated lung cancer who were referred to our bronchoscopy unit after suspicion of locoregional recurrence from January 2006 to October 2014. Recurrence suspicion was based on hilar or mediastinal lymph node enlargement on CT scan (>10 mm in the short axis on CT) and/or abnormal nodal fludeoxyglucose (FDG) avidity on PET-CT during follow up with or without pulmonary node/s or mass/es. The medical records of all patients were reviewed and clinical characteristics were introduced in a database.

### EBUS-TBNA

EBUS was performed at an out-patient setting using a flexible bronchoscope [BFUC180F, Olympus Optical Co Ltd., Tokyo, Japan] with a distal probe capable of producing linear parallel scans of both mediastinal and peribronchial tissues also a working channel suited for the performance of TBNA under direct ultrasound guidance. Local anesthesia and sedation were achieved using topical lidocaine spray and intravenous midazolam, propofol and/or fentanyl in accordance with the standard recommendations [19]. Identified mediastinal and lobar nodes with short-axis diameter of 5 mm or more were targeted under direct ultrasound visualization with a 22-gauge cytology needle specially designed for EBUS-TBNA [NA-201SX-4022, Olympus Optical Co Ltd.]. After passing through the bronchoscope channel, the needle was pushed out of the sheath and inserted into the tracheal or bronchial wall under ultrasound guidance. At the target tissue, the needle tip was located, and then it was pushed forth and back with application of negative pressure using a syringe (with 10-mL suction) connected to the proximal end of the catheter. Finally, the suction was ceased while withdrawing the needle out of the target structure. Samples were labeled according to their origin, whether it was a normal node showing lymphocytic cells and no neoplastic cells, or a metastatic node showing neoplastic cells. Aspirates containing only isolated dysplastic, bronchial, esophageal or blood cells *or* necrotic tissue were considered inadequate. Nodal sampling was targeted and nodes with high suspicion of malignancy (enlarged on CT scan and/or abnormal FDG avidity) were firstly sampled. If rapid on site examination confirmed malignancy the procedure was then finished. In case of a non malignant result, suspicious nodes were sampled three times before ruling out malignancy and a complete systematic sampling (including both lower paratracheal, and subcarinal stations) was performed.

### EUS-B-FNA

In cases with lesions that were inaccessible through EBUS-TBNA (nodal stations 5, 8 or 9) the patient underwent directly EUS-B-FNA. In cases with lesions accessible through EBUS-TBNA the patient firstly underwent EBUS-TBNA. If the lesion was partially visible through EBUS-TBNA and located in a station accessible through EUS-B-FNA then the patient underwent EUS-B-FNA in a single-session procedure. EUS-B-FNA was performed guiding the bronchoscope through the pharynx and advanced into the esophagus under gentle pressure. The sampling method did not differ to that previously described for EBUS-TBNA.

### Pathology

The aspirated material in the needle was recovered and the specimens were placed on slides and fixed with 95% ethanol. The slides were stained for one minute with haematoxylin for rapid on-site evaluation. Papanicolau staining with orange A and eosin was done later in the pathology laboratory. The cytologist classified satisfactory nodal samples as “normal tissue negative for malignancy” when the sample contained 40 lymphocytes per high-power field in cellular areas of the smear and/or clusters of pigmented macrophages and no neoplastic cells, or as “metastatic” when recognizable groups of malignant cells were present [20]. Nodes containing only isolated dysplastic, bronchial, esophageal or blood cells or necrotic tissue were considered as non-representative of the targeted structure, and were classified as inadequate. Cell blocks were obtained and processed from the specimens recovered whenever extra material was available after the preparation of a minimum of four slides.

### Definitions

Cases in which EBUS-TBNA demonstrated malignant nodes with the same histology as the previous treated lung cancer were considered as recurrence (true positive) and no confirmatory tests were required. Cases in which EBUS-TBNA demonstrated specific benign diseases were considered as true negative and no confirmatory tests were carried out. Cases where EBUS-TBNA demonstrated lymphocytes without malignant cells (negative EBUS-TBNA) underwent confirmatory surgery as a “gold standard” or radiological follow up. Negative EBUS-TBNA was considered true negative if the surgical procedures did not demonstrate nodal malignancy or if remained stable during radiological follow up for 12 months. Negative EBUS-TBNA were considered false negative if the surgical procedures demonstrated nodal malignancy or if radiological progression was proved by CT. Patients that presented with extrathoracic progression that required chemotherapy and/or could not complete 12 months of radiological follow up were also considered false negative. Although diagnostic yield of EBUS-TBNA for global malignancy included patients with metachronous tumours, outcomes of these patients were not used in evaluating diagnostic yield of EBUS-TBNA for locoregional recurrence. Metachronous tumours were defined by Martini et al. [21] as follows: 1) Different histological type from the primary tumour or 2) Same histological type if: a) Free interval between tumours is at least 2 years or b) Origin from carcinoma in situ or c) Second cancer in different lobe or lung, but: i) No carcinoma in lymphatics common to both, ii) No extrapulmonary metastases at time of diagnosis.

### Statistical analysis

Data was entered into a database and analyzed using SPSS software, version 18.0 [Chicago, IL, USA]. Categorical variables were expressed as absolute and relative frequencies, continuous variables as means and standard deviations (SD) and non-normally distributed data as medians and interquartile ranges (IQR). The diagnostic sensitivity, specificity, positive predictive value (PPV), negative predictive value (NPV) and overall accuracy of EBUS-TBNA were calculated according to standard definitions for diagnosis of both locoregional recurrence and global malignancies, including metachronous tumors, in patients with previous surgically-treated lung cancer.

## Results

Seventy-three patients were included. Patient characteristics are shown at Table 1. Previous histological subtypes of lung cancer were predominantly squamous cell-carcinoma and adenocarcinoma and the majority of patients had undergone previous lobectomy. Most of the patients had a previous systematic mediastinal nodal dissection; the median number of dissected nodes was 14.5 per patient. Six patients had a previous staging mediastinoscopy and 20 patients had undergone EBUS-TBNA before surgery. Primary therapy for lung cancer included surgical resection alone in 47 patients; surgical resection followed by chemotherapy in 16; surgical resection followed by radiotherapy in one patient and induction chemo radiotherapy followed by surgical resection in 9 patients. One patient was diagnosed as stage IV but could benefit for surgical treatment after resection of the single extrathoracic metastasis. The median time between primary surgical treatment and recurrence suspicion was 23 months. CT findings were mediastinal nodal enlargement alone in 50 patients and mediastinal nodal enlargement with lung nodes/masses in 23 patients.

**Table 1:** Patients’ characteristics

Gender: Male (67/73 (91.7%))	
Age: Mean 69 (SD ± 10.4).	
Time to recurrence: 23 (IQR: 11.5–49).	
CT findings:	
Isolated mediastinal lymphadenopaties: 50 (68.5%)	
Mediastinal lymphadenopaties & lung nodules/masses: 23 (31.5%)	
Stage:	
IA: 26 (35.6%)	
IB: 17 (23.3%)	
IIA: 12 (16.4%)	
IIB: 13 (17.8%)	
IIIA: 4 (5.5%)	
IV: 1 (1.4%)	
Surgical treatment:	
Lobectomy 48 (65.7%)	
Bilobectomy 3 (4.1%)	
Neumonectomy 8 (11%)	
Wedge resection 14 (19.2%)	
Systematic mediastinal nodal dissection:	
Linfadenectomy: 58 (79.5%)	
Nodes diseccted: 14.5 (IQR: 11–20.75)	
Stations diseccted: 4.5 (±1)	
Previous mediastinoscopy: 6 (8.2%)	
Previous EBUS: 20 (27.4%)	
Previous treatment:	
Surgery 47 (64.4%)	
Adyuvant chemotherapy 16 (21.9%)	
Adyuvant radiotherapy 1 (1.4%)	
Trimodal treatment 9 (12.3%)	

**Fig. 1:**
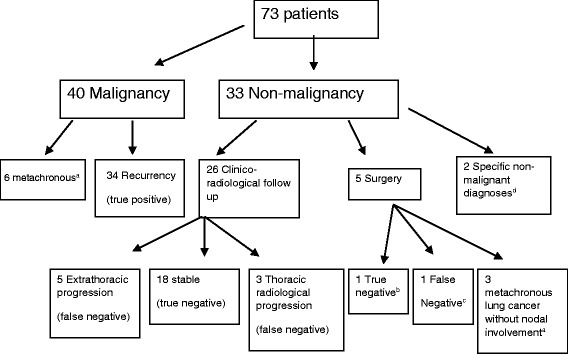
^a^Excluded for analysis of recurrence. ^b^Mediastinoscopy showing normal lymph tissue. ^c^One patient with malignant N1 interlobar node in the resection specimen. ^d^One tuberculosis, one foreign body reaction

EBUS-TBNA confirmed malignancy in 40 patients while in the remaining 33 did not show malignancy (Fig. 1). The entire 40 patient with malignant nodes were presented with the same histological type as the previous treated lung cancer. However, six patients were considered as metachronous disease instead of recurrence. Of the 34 patients confirmed to have recurrence by EBUS-TBNA, 15 patients underwent chemotherapy, 3 radiotherapy, 14 concurrent chemoradiotherapy and 2 patients received best supportive care. In most of the cases, nodal recurrence affected ipsilateral hilar or mediastinal nodes and, in 20% recurrence affected contralateral mediastinum (N3). Eleven malignant lymph nodes (8 hilars, 3 paraesophageal stations), collected from 10 patients who resembled 29.4% of all recurrent patients diagnosed by EBUS-TBNA, were out of the reach of mediastinoscopy.

Of the 33 patients with non-malignant EBUS-TBNA, two had a specific non-malignant disease diagnosed by EBUS-TBNA (1 nodal tuberculosis, 1 foreign body reaction). Of the other 31 patients, 26 patients underwent radiological follow up and 5 patients underwent surgery. Of the 5 patients that underwent surgery one patient with isolated lymphadenopathies underwent mediastinoscopy which showed normal lymph tissue and thereby was considered as true negative; the other 4 patients that presented node or mass associated to the nodal enlargement underwent lobectomy: 3 patients had a final diagnosis of metachronous lung cancer without nodal involvement while one other patient presented a N1 positive interlobar node in the lobectomy resection sample and thus was considered as a false negative of the EBUS-TBNA.

Of the 26 patients who had radiological follow up 18 patients remained stable after 12 months, three presented with thoracic radiological progression and 5 patients presented with extrathoracic progression and died before completing 12 months of follow-up and thereby were considered false negative.

**Fig. 2:**
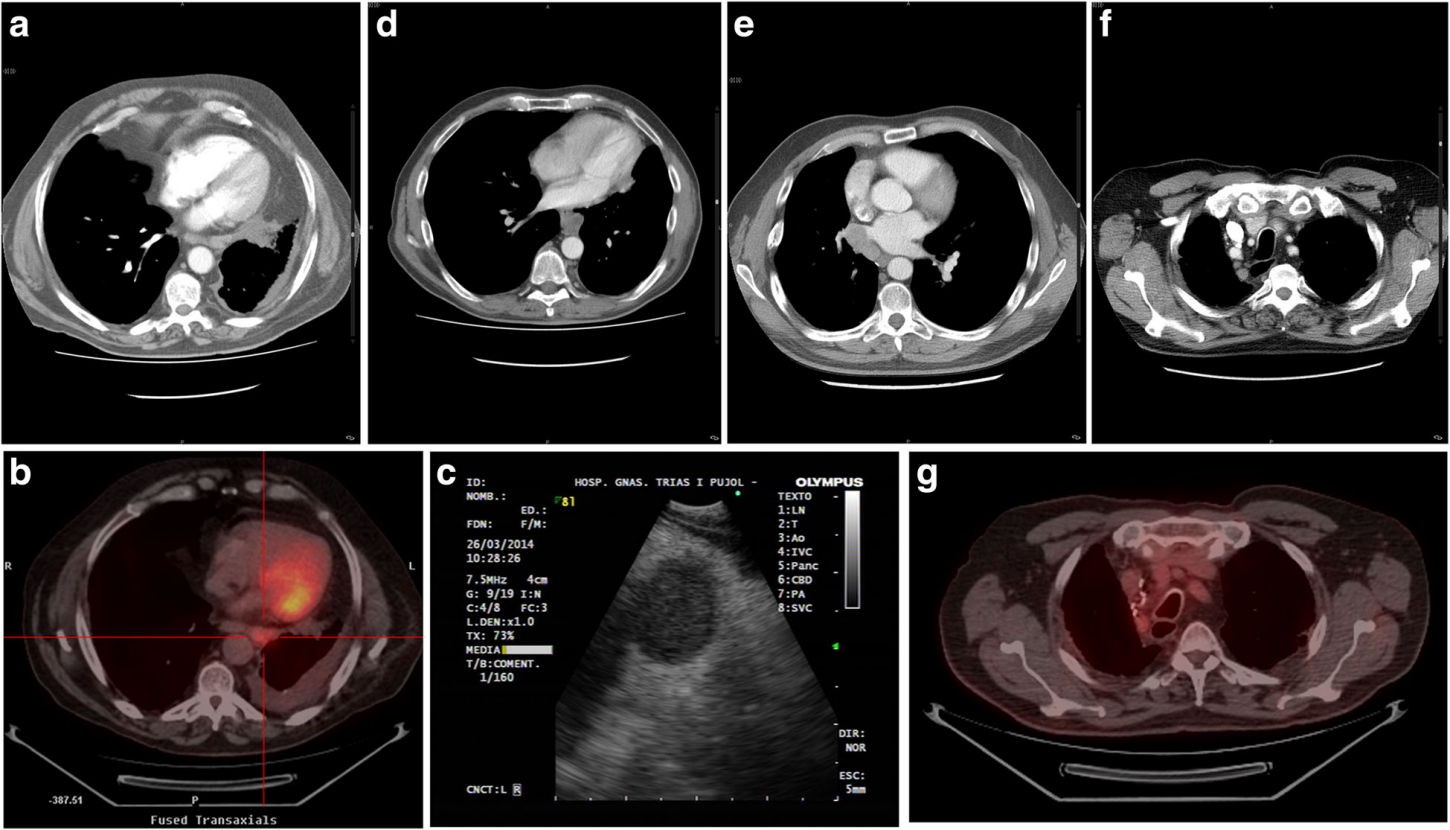
Four cases of recurrence diagnosed by EUS-B-FNA: *Case 1* (**a**, **b**, **c**): a 68-years old man with a stage IIA squamous-cell carcinoma in LLL treated by means of lobectomy presented a pleural effusion on the first CT control 6 months after the surgery, the PET/CT showed a high uptake on a 8 left station node, an EUS-B-FNA confirmed recurrence. *Case 2* (**d**): a 55-years old man with a previous stage IA NSCLC-NOS in RUL treated by means of wedge resection presented an 8 left lymphadenopathy on the first CT control 6 months after the surgery. *Case 3* (**e**): a 40-years old man with a carcinoid on middle lobe treated by means of RLL and ML bilobectomy presented a mass/nodal enlargement on right 8 station that invaded the pulmonary veins, the diagnosis was consistent with atypical carcinoid. *Case 4* (**f**, **g**): a 71-year old man with a stage IIA adenocarcinoma in RUL treated by means of lobectomy presented a 2R adenopathy in a CT 21 months before the surgery. Although being a paratracheal node the approach was easier through the esophagus

Seven patients underwent EUS-B-FNA because the lesion was difficult to be accessed (one 2R station node, one 4 L station node) or inaccessible for EBUS (five paraesophageal (8) stations) (Table 2, Fig. 2). Of the 2 patients with a lesion accessible by means of EBUS-TBNA both had an EBUS-TBNA before the EUS-B-FNA in a single-session procedure while the 5 patients with lesions not reachable by means of EBUS-TBNA directly underwent EUS-B-FNA. EUS-B-FNA confirmed the recurrence in four cases. There were no major complications related to EBUS-TBNA.

**Table 2:** EBUS procedure

Total nodes sampled: 213	
Nodes sampled (per patient): 2.92 (SD ± 2.2)	
Stations sampled (per patient): 2 (SD ± 1.2)	
Malignant nodes^a^: 45	
Mediastinal: 37	
2 L: 1	2R: 1
4R: 7	4 L: 11
7: 14	
8 L: 2	8 L: 1
Hilar: 8	
10 L: 1	10R: 2
11 L: 2	11R: 3
Mean size^b^: 14.8 (IQR: 11.5–18.4)	
Esophagus (EUS-B-FNA): 7 (9.6%)	
N in recurrence (34):	
N1: 5 (14.7%)	
N2: 22 (64.7%)	
N3: 7 (20.6%)	

^a^In patients with recurrence diagnosed by EBUS-TBNA

^b^In mm (short-axis diameter)

The sensitivity, specificity, negative predictive value, positive predictive value and overall accuracy of EBUS-TBNA for the diagnosis of locoregional recurrence in patients with previous surgically-treated lung cancer were 80.9, 100, 69.2, 100 and 86.6% respectively. The sensitivity, specificity, negative predictive value, positive predictive value and overall accuracy of EBUS-TBNA for the diagnosis of global malignancy, including metachronous tumours, in patients with previous surgically-treated lung cancer were 81.6, 100, 72.7, 100 and 87.6% respectively.

## Discussion

Locoregional recurrence represents a significant problem in lung cancer, which corresponds to a quarter of recurrences after surgery [22]. Reported rates of locoregional recurrence of NSCLC after surgery vary widely in literature due to the heterogeneity of the studies, as some of them including small sample sizes, variable disease stages, differences in follow up time and unclear definitions of recurrence versus metachronous tumours. Locoregional recurrences typically occur rapidly, as nearly 90–95% of all local recurrences develop during the first five years after the initial surgery [23]. Pathological stage, surgical technique (wedge resection or segmentectomy versus full lobar resection) and patients’ characteristics have been demonstrated to be independent predictors of recurrence. Although postoperative surveillance programs have not established a survival benefit, most guidelines recommend follow up of patients after curative-intent surgery. In our series, half of the patients were presented with nodal local recurrence, 6.8% extrathoracic metastases and 12% metachronous lung cancer, confirming that the likelihood of malignancy in these patients is high and thus the diagnosis and clinical-decision making cannot rely only on image-based explorations.

Before EBUS-TBNA, mediastinoscopy was the most used diagnostic method as an invasive approach to explore the mediastinum. However, few studies have focused on the role of mediastinoscopy in the diagnosis of locoregional recurrence. The concept of “complex” mediastinum refers to an altered fibrotic mediastinum secondary to previous thoracic surgery (primary mediastinoscopy or nodal dissection) or induction therapy [24]. Although some studies performed by expert surgeons in referral hospitals have shown good accuracy of remediastinoscopy, with complication rates similar to primary mediastinoscopy [25], remediastinoscopy is not widely used due to concerns about safety and usefulness [26]. In our series, one third of the patients had concurrent radiotherapy and/or chemotherapy and 7 patients had a previous mediastinoscopy. Regarding the diagnosis of local lymph node recurrence, only one patient required mediastinoscopy after an EBUS. This particular patient neither underwent previous mediastinoscopy nor received neo/adjuvant therapy. The mediastinoscopy showed no malignancy and the patient remained stable in subsequent radiological follow up. In our series, almost one third of the patients with recurrence confirmed by EBUS-TBNA presented with nodes out of the reach of mediastinoscopy: 3 patients presented with paraesophageal lesions and 7 patients had hilar lesions.

One of the advantages of EBUS-TBNA is its ability to sample hilar nodes. Probably this advantage has not been properly emphasized. One possible explanation is that EBUS-TBNA has been mainly used for mediastinal staging of lung cancer where hilar involvement is not relevant enough since contralateral hilar N3 involvement without N3 mediastinal affection is not very frequent and the distinction between N0/N1, although has importance in the prognosis and the choice of treatment, rarely affects the surgical indication. Nevertheless, the evaluation of N1 nodes is crucial in patients undergoing sublobar resection or local tissue-sparing treatments such as brachytherapy, radiofrequency ablation and stereotactic body radiation. These are the only therapeutic options to many patients who cannot benefit from conventional surgical intervention, including those with previous surgical treatment like in our series. In our study the only surgical false negative case presented with N1 interlobar node. The reported sensibility for EBUS-TBNA in the diagnosis of N1 disease is lower than that described for N2 staging [27], mainly because the size of the convex probe EBUS in many cases does not permit to reach N1 nodes beyond station 11. However, this issue could be solved with the development of new thinner convex probe EBUS. Recently, Wada et al. [28] described their first experience with a new thin convex probe echobronchoscope [BF-Y0046 Olympus Medical Systems Corp] with a thinner tip (5.9 mm) and a larger bending angle than conventional convex probe ecobronchoscopes. These authors reported an improved accessibility to the distal airways in a porcine model, suggesting that thinner echobronchoscopes would increase the diagnostic yield of N1 nodes (especially those beyond the hilum) and peripheral nodes/masses.

Endoscopic ultrasound with a convex probe ultrasonic bronchoscope (EUS-B-FNA) was first described in 2009 [10]. EUS-B-FNA broads the diagnostic yield of EBUS-TBNA alone because it allows the approach of nodal stations that are not in contact with the tracheobronchial wall (stations 5 and lower mediastinal stations: 8, 9) and also increases the access to nodes that could be better achieved through the esophagus. Moreover EUS-B-FNA is also better tolerated and safer in patients with respiratory impairment. Although these advantages, EUS-B-FNA requires a learning period before systematically employed, as the endobronchial references are lost on the white light image and the ultrasonographic anatomy boundaries vary from the endobronchial view. In our series, ten percent of the patients underwent EUS-B-FNA, and 4 patients (12.9%) with recurrence diagnosed by EBUS-TBNA were exclusively diagnosed by EUS-B-FNA (3 in paraesophageal stations and one in 2R station). In our study, some nodes although located on subcarinal or paratracheal stations, were more easily reached by the esophagus (Fig. 2) due the post-surgical changes in the mediastinal architecture. Few studies have described the usefulness of EUS-B-FNA apart from lung cancer staging. Szlubowski et al. [11] published their experience with EUS-B-FNA combined with EBUS-TBNA in the lung cancer restaging after induction therapy and other two studies described the usefulness of EUS-B-FNA in the sampling of left adrenal gland in patients with lung cancer [12, 13]. To our knowledge this is the first study in which EUS-B-FNA has been used in the diagnosis of locoregional lung cancer recurrence.

Although the interpretation of the visualized mediastinal structures after surgery is more difficult than in a naïve mediastinum, our study demonstrates excellent diagnostic performance of EBUS-TBNA in the diagnosis of locoregional recurrence of patients with surgically treated lung cancer. Our results are similar to those reported for new developed lung cancer staging and also comparable to those to previous studies that included shorter series of surgically-treated patients. Yamamoto et al. [17] in a series of 40 surgically-treated patients showed a sensitivity and NPV of 100%, while Han et al. [16] in a series of 42 surgically-treated patients showed a sensitivity of 94.3% and a NPV of 77.8%. Other studies that included non-surgically-treated patients [14, 15, 18] also demonstrated a high diagnostic accuracy of EBUS-TBNA in the diagnosis of lung cancer recurrence. Thus, changes in the mediastinal anatomy in a post-surgical mediastinum do not affect the diagnostic yield of EBUS-TBNA procedure. Remarkably, in another situation of “complex” mediastinum, following induction therapy, EBUS-TBNA showed lower diagnostic value in restaging of lung cancer [29, 30]. This is probably not due to the procedure itself, but because of complexity in the interpretation of the pathological samples. It has been previously reported that aspirates from malignant nodes treated with chemotherapy may content less cellular burden that could have necrotic tissue, making the pathologic interpretation more difficult.

## Conclusions

EBUS-TBNA is proved to be an accurate, safe and minimally invasive procedure in the diagnosis of locoregional recurrence of surgically-treated lung cancer and should be considered as a first choice in patients with radiological abnormalities during follow up. EUS-B-FNA demonstrates to be useful in patients with locoregional lung cancer recurrence, not only in stations not reachable by EBUS-TBNA, but also in paratracheal nodes that could be better attained by the esophagus, whenever there are changes in the normal mediastinal architecture.

## Abbrevations

COPD: Chronic obstructive pulmonary disease
CT: Computed tomography
EBUS-TBNA: Endobronchial ultrasound transbronchial-guided needle aspiration
EUS-B-FNA: Endoscopic ultrasound fine-needle aspiration using an echobronchoscope
EUS-FNA: Endoscopic ultrasound fine-needle aspiration
FDG: Fludeoxyglucose
IQR: Interquartilic range
NPV: Negative predictive value
NSCLC: Non-small cell lung cancer
PET/CT: Positron emission tomography/Computed tomography
PPV: Positive predictive value
SD: Standard deviation
